# Natural 4-Hydroxy-2,5-dimethyl-3(2*H*)-furanone (Furaneol^®^)

**DOI:** 10.3390/molecules18066936

**Published:** 2013-06-13

**Authors:** Wilfried Schwab

**Affiliations:** Biotechnology of Natural Products, Technische Universität München, Liesel-Beckmann-Str. 1, 85354 Freising, Germany; E-Mail: Wilfried.Schwab@tum.de; Tel.: +49-8161-71-2912; Fax: +49-8161-71-2950

**Keywords:** 3(2*H*)-furanone, biosynthesis, Maillard reaction, D-fructose-1,6-diphosphate

## Abstract

4-Hydroxy-2,5-dimethyl-3(2*H*)-furanone (HDMF, furaneol^®^) and its methyl ether 2,5-dimethyl-4-methoxy-3(2*H*)-furanone (DMMF) are import aroma chemicals and are considered key flavor compounds in many fruit. Due to their attractive sensory properties they are highly appreciated by the food industry. In fruits 2,5-dimethyl-3(2*H*)-furanones are synthesized by a series of enzymatic steps whereas HDMF is also a product of the Maillard reaction. Numerous methods for the synthetic preparation of these compounds have been published and are applied by industry, but for the development of a biotechnological process the knowledge and availability of biosynthetic enzymes are required. During the last years substantial progress has been made in the elucidation of the biological pathway leading to HDMF and DMMF. This review summarizes the latest advances in this field.

## 1. Introduction

**Figure 1:**
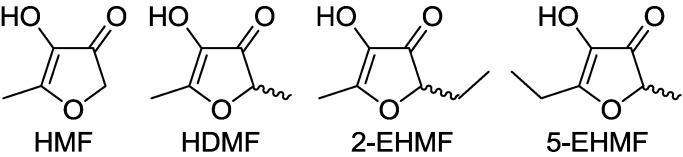
Structures of 3(2*H*)-furanones: 4-hydroxy-5-methyl-3(2*H*)-furanone (HMF), 4-hydroxy-2,5-dimethyl-3(2*H*)-furanone (HDMF) and the tautomers 2-ethyl-4-hydroxy-5-methyl-3(2*H*)-furanone (2-EHMF) as well as 5-ethyl-4-hydroxy-2-methyl-3(2*H*)-furanone (5-EHMF).

In 1960, 4-hydroxy-2,5-dimethyl-3(2H)-furanone (Furaneol^®^, HDMF, also 4-hydroxy-2,5-dimethyl-2,3-dihydrofuran-3-one) was reported for the first time as a product of the Maillard reaction or non-enzymatic browning [1]. Maillard products result from a complex series of chemical reactions between an amino acid and a reducing sugar, usually requiring heat [2,3]. Although the reactions have been analyzed since 1912 and several of the routes and intermediates have been postulated many are still unknown. The best known products that arise from C5, C6 and C7 sugars via Amadori product intermediates are 4-hydroxy-5-methyl-3(2*H*)-furanone (HMF), HDMF and the tautomers 5-(or 2)-ethyl-4-hydroxy-2-(or 5)-methyl-3(2*H*)-furanone (EHMF), respectively (Figure 1). Alternatively, HDMF is formed by addition of a C1 fragment, derived from glycine by Strecker degradation, to a diketose derived from a C5 carbohydrate. EHMF may arise similarly with addition of a C2 fragment produced from alanine to a C5 diketose [4]. The Amadori products, the diketose intermediates and the furanones have been identified in a multitude of processed food. The levels of furanones produced depend on the type and amount of sugars and amino acids available in the raw food material, the pH value and the heating regime. Besides their chemical formation during thermal treatment of food 4-hydroxy-3(2*H*)-furanones are biosynthesized by plants [5], microorganism, and insects although the detailed formation pathways are still unknown [4].

Due to their low odor thresholds and attractive odor qualities 4-hydroxy-3(2H)-furanones are considered exceptional aroma compounds and are used, among others, for the flavoring of jams, jellies, beverages, ice creams, alcoholic drinks and sweets. Odor thresholds ranging from 0.03 to 1,700 µg/L, 2,100 to 23,000 µg/L and 0.04 to 21 µg/L have been reported for HDMF, HMF and EHMF, respectively [6]. Their caramel-like flavor is associated with a planar enol-oxo group of a cyclic dicarbonyl derivative able to form strong hydrogen bonds.

Literature about the history, occurrence, synthesis, analysis, property, stability, flavor characteristics, biosynthesis, bioactivity and toxicology of 4-hydroxy-3(2H)-furanones has been summarized in two comprehensive reviews [4,6]. This article will only outline the recent advances in these fields.

## 2. Properties

The stability of HDMF, its methoxy derivative 2,5-dimethyl-4-methoxy-3(2H)-furanone (DMMF), and the naturally occurring glycosidically bound forms HDMF β-D-glucoside and HDMF 6′-*O*-malonyl-β-D-glucoside [7,8] was investigated at different pH values [9]. Only slight decomposition of DMMF and HDMF β-D-glucoside was observed, whereas HDMF and HDMF 6′-*O*-malonyl-β-D-glucoside were found to be unstable at all pH values. In contrast to the rapid hydrolysis of HDMF β-D-glucoside, the malonylated glucoside remained unaffected by enzymatic treatment with β-glucosidase [9].

**Figure 2:**
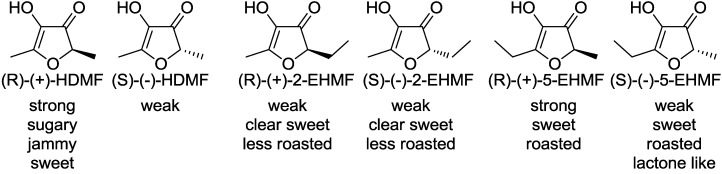
Absolute configuration of 2-substituted 3(2H)-furanones and their odor perceptions.

Although significant organoleptic differences were perceived between the enantiomers of optically active 2-substituted-3(2*H*)-furanones, the absolute configurations of the furanone derivatives have remained ambiguous for the past 40 years. Only recently, the absolute configurations of HDMF and EHMF were unraveled for the first time using vibrational circular dichroism as well as chemical relay reactions [10,11,12]. Odor evaluation of each enantiomer revealed relationships between their configurations and their odor activities (Figure 2).

In plants HDMF is stabilized by glucosylation via an as yet unknown glucosyltransferase [13]. Biomimetic enzymatic synthesis of 3(2*H*)-furanone glucosides were achieved by sucrose phosphorylase [14]. HDMF was converted to its 4-*O*-α-D-glucopyranoside, whereas EHMF yielded two major products, namely 2-ethyl-5-methyl-3(2*H*)-furanone and 5-ethyl-2-methyl-3(2*H*)-furanone 4-*O*-α-D-glucopyranoside. The glucosides are more stable than their aglycones, but can be hydrolyzed by an intestinal acetone powder from pigs [14].

One of the main shortcomings of the Maillard reaction is the lack of ways to control the different pathways, particularly when it is desired to direct the reaction away from the formation of toxic products to more aroma and color generation. Thus, systematic approaches were conducted, varying numerous reaction parameters, to model the kinetics of the reaction. These studies indicated that L-rhamnose is an excellent precursor of HDMF yielding more than 40 mol% [15,16], whereas the use of specifically phosphorylated carbohydrates may impart some elements of control over the aroma profile generated by the Maillard reaction [17]. Besides, it was suggested that HDMF is formed from methylglyoxal and D-glucose by different pathways [18].

## 3. Physiological Activity and Toxicology

**Scheme 1:**
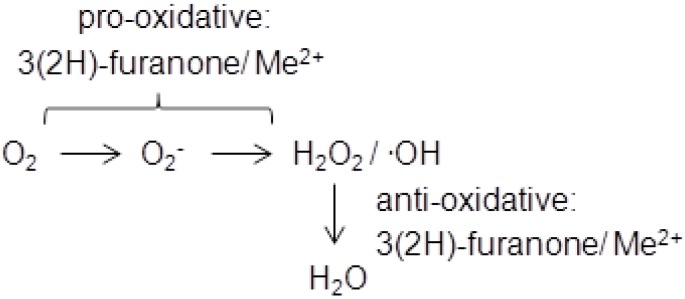
3(2*H*)-Furanones function as pro- and as anti-oxidants. Me^2+^ transition metal cation, O_2_^−^ superoxide radical anion, H_2_O_2_ hydrogen peroxide,**^.^**OH hydroxyl radical.

During the last decade the cytotoxicity of 3(2*H*)-furanones has attracted much attention and has been intensively investigated. DNA-breaking activity was demonstrated first for HMF and later also for HDMF and EHMF [19]. The 3(2*H*)-furanone/transition metal–mediated generation of reactive oxygen species was held responsible for DNA strand breaks and the formation of 8-hydroxy-2′-deoxyguanosine [20]. The 3(2*H*)-furanones, which are known pro-oxidants in foods [21], can produce superoxide radicals through, e.g., the reduction of cupric ion to cuprous ion, resulting in the conversion to hydrogen peroxide and hydroxyl radicals [22]. Interestingly, the related 2(5*H*)-furanones were inactive [20]. In contrast, the inhibition of cataract formation by furanones in spontaneous cataract rat demonstrated an anti-oxidative function of HDMF [23]. It was concluded that the protective activity of HDMF and EHMF against superoxide radicals in lens tissue contributed to inhibiting the onset of spontaneous cataracts [24]. Thus, 3(2*H*)-furanones feature anti-oxidative as well as pro-oxidative properties depend on the availability of oxygen species which is in line with the instability of HDMF (Scheme 1).

Bioavailability and metabolism of HDMF was determined in humans. HDMF was administered to four male and two female volunteers using fresh strawberry fruit as natural furanone source [25]. Female and male volunteers excreted 81–94% and 59–69%, respectively of the HDMF dose (total of free and glycosidically bound HDMF) as HDMF glucuronide. HDMF, HDMF β-D-glucoside and its 6’-O-malonyl derivative were not detected in human urine. Absorption of HDMF and EDHF was also analyzed in mice after a single intraperitoneal or oral administration of 0.5–1.0 g kg^−1^ [26]. They appeared in plasma 5 min after oral application, reached maximum between 15–45 min, and gradually disappeared after 2 h, indicating that they were absorbed by the digestive tract. HDMF and EDHF induced micronucleated reticulocytes in a dose-dependent manner and thus caused genetic damage after oral administration. Intestinal absorption of furanones and metabolic conversion was also examined by using Caco-2 cell monolayers [27]. The transport of 3(2*H*)-furanones could not be saturated even at levels of 500 µM and occurred in both directions. Passive diffusion by paracellular transport was proposed.

The effect of long-term supplementation of HDMF on mouse lipid peroxidation and type I and IV allergy responses was investigated [28]. Levels of thiobarbituric acid-reactive substances in lung were remarkably increased, and those in kidney and liver were slightly decreased by supplementation of HDMF, whereas allergen-sensitized type I and IV allergy responses of mice may be enhanced. However, a study investigating the effect of EDHF on oxidative damage of erythrocytes and low density lipoprotein (LDL) indicated that EDHF protected human erythrocytes membranes and LDL against iron ion-induced oxidative modifications [29].

The de-pigmenting capacity of HDMF was investigated in B16 melanoma cells [30]. The melanin content and tyrosinase activity induced by α-melanocyte-stimulating hormone were inhibited significantly. The study showed that HDMF suppressed the phosphorylation of cAMP response element binding protein, which is induced by protein kinase A and suggested that HDMF may be an effective inhibitor of hyperpigmentation.

HDMF also exhibited broad spectrum antimicrobial activity in an energy-dependent manner without hemolytic effect on human erythrocytes [31]. Cell cycle analysis showed that HDMF arrested the cell cycle at the S and G2/M phase in *Candida albicans* and thus, HDMF may have potential as an anti-infective agent in human microbial infections.

## 4. Occurrence

Since its discovery in pineapples in 1965, HDMF and its metabolites [2,5-dimethyl-4-methoxy-3(2*H*)-furanone (DMMF), HDMF-β-D-glucopyranoside (HDMF-glucoside) and HDMF-6′-malonyl-β-D-glucopyranoside (HDMF-glucoside-malonate)] have been isolated as natural products from many different fresh fruits such as strawberry [32], raspberry [33,34], tomato [35], kiwi [36], lychee [37], and snake fruit [38]. The natural sources of HDMF have been reviewed [4,6]. By application of aroma extract dilution analysis and calculation of odor activity values, HDMF was defined as one of the key odorants in pineapples [39,40]. HDMF was also among the volatiles that showed higher odor units in more preferred tomato cultivars [41]. Highest HDMF concentrations were found in the summer crop of home-grown tomatoes while the lowest levels were detected in the common ethylene-ripened, field-grown, supermarket tomatoes [35]. Levels of HDMF appeared to increase in strawberry fruit after 9 days of treatment with methyl jasmonate [42]. 

HDMF has been frequently found in wines [37,43]. Generally, its concentrations in white wines were lower than in red wines [44]. Results showed a significant variation in the level of HDMF in wines with grape variety demonstrating the biosynthesis of the furanone in grape berries. However, the study of the volatile composition of oak wood pieces that are used as alternatives to barrels for aging wines revealed high levels of HDMF thus, wood pieces might serve as an additional source for the 3(2H)-furanone [45]. HDMF has been identified as one of the key odorants in Spanish aged red wines and Aragonez clonal musts by GC-olfactometry [46,47,48] and in Bordeaux red wines by aroma extract dilution analysis [49]. It can still be detected after 5–6 years of storage [50] and has been found to be one of the components that distinguish different high-quality Spanish aged red wines [51]. Importantly, a synergistic effect of HDMF and EDMF was observed in rosé wine which had a significant impact on fruity and caramel notes [52]. 

HDMF also contributes to the aroma of human breast milk [53] and to milk products such as nonfat dry milk [54] and sweet whey powder [55]. In the last two products it is probably chemically formed during the manufacturing process. Consistently, HDMF levels increased during conching, a process used to turn cacao into chocolate [56]. In cheeses, it appears that furanones are rather biochemically synthesized during ripening [57] similar to soy sauce which also runs through a fermentation process [58]. In thermally generated imitation flavors produced from soybean-based enzyme-hydrolyzed vegetable protein HDMF is primarily formed by the Maillard reaction [59]. Similarly, furanones are chemically formed from α-dicarbonyl intermediates during storage of Pilsner beer [60] and were detected as Maillard products in country ham [61] and cooked beef [62].

## 5. Microbial Formation

### 5.1. Yeast

HEMF was isolated from fermented soy sauce for the first time as it is the major component of the flavor of soy sauce [4,6]. The formation of HEMF was promoted by cultivating the halo-tolerant yeast, *Zygosaccharomyces rouxii*, in a medium including the amino-carbonyl (Maillard) reaction products based on ribose and glycine [63]. The mechanism was investigated by stable isotopes of the corresponding compounds. The skeleton of the five-membered ring and the methyl group of the side chain of HEMF originated from ribose, and the ethyl group was derived from D-glucose or acetaldehyde. The role of the yeast in HEMF formation was not only to provide the D-glucose metabolite (acetaldehyde), but also in combining the Maillard reactants with the D-glucose metabolite [64].

Formation of HMF was demonstrated in cytosolic extracts from *Z. rouxii* after incubation with a number of carbohydrate phosphates [65]. Since HMF was spontaneously formed from ribulose-5-phosphate via the Maillard intermediate 4,5-dihydroxy-2,3-pentanedione [66], it was assumed that ribulose-5-phosphate is enzymatically generated in cytosolic extracts and then chemically transformed to HMF. The hypothesis was confirmed by the production of HMF in mixtures containing commercially available enzymes and isotopically labeled D-glucose-6-phosphate. Interestingly, HMF has been identified as extracellular signal molecule Al-2 catalyzed by the enzyme LuxS and functions in cell-to-cell communication in bacteria [67]. The chemical formation of Al-2 from ribulose-5-phosphate may also occur *in vivo* and may be responsible for the Al-2-like activities reported for organisms lacking the luxS gene [68].

The formation of HDMF by *Z. rouxii* from D-fructose-1,6-diphosphate was studied under various culture conditions [69,70]. Growth of *Z. rouxii* and formation of HDMF was not observed when D-fructose-1,6-diphosphate served as sole carbon source. Although *Z. rouxii* cells grew in media containing D-glucose as the sole carbon source, HDMF was only produced when D-fructose-1,6-diphosphate was added. The HDMF levels always correlated with the yeast cell count and D-fructose-1,6-diphosphate concentration. Only single labeled HDMF was formed after addition of 1-^13^C-D-fructose-1,6-diphosphate but unlabeled furanone was formed in the presence of ^13^C_6_-D-glucose. Thus, the carbons of HDMF originate exclusively from exogenously supplied D-fructose-1,6-diphosphate [69]. Higher pH values of the medium had a positive effect on HDMF formation but retarded cell growth resulting in an optimal pH value of 5.1. Salt stress stimulated HDMF production. Addition of o-phenylenediamine, a trapping reagent for α-dicarbonyl (Maillard) intermediates, to the culture medium revealed the formation of three quinoxaline derivatives derived from D-fructose-1,6-diphosphate [71]. Identification of the structures demonstrated for the first time the chemical formation of 1-deoxy-2,3-hexodiulose-6-phosphate, a generally expected but never identified intermediate in the formation pathway of HDMF. Additional enzymatic steps were assumed since HDMF was detected only in the presence of *Z. rouxii* cells. HDMF is also chemically formed in solutions containing D-fructose-1,6-diphosphate and NAD(P)H at ambient temperature [72]. The NAD(P)H was mandatory and application of labeled precursors indicated a hydride transfer to C-5 or C-6 of the D-fructose-1,6-diphosphate skeleton. It seems that the biological and chemical formation of HDMF from D-fructose-1,6-diphosphate follow similar pathways.

Optically active natural products exhibit characteristic enantiomeric excesses due to stereoselective, enzymatically catalyzed reactions during their biosynthesis. Although the enzymatic generation of HDMF by *Z. rouxii* and fruits is anticipated, the naturally occurring compound is racemic. The rapid racemization of HDMF due to keto-enol-tautomerism accounts for this observation [73,74]. ^1^H-NMR analysis tracing the exchange of the proton bound to the furanone-ring at C-2 with deuteron and chiral phase capillary electrophoresis analysis showed that the racemization rate of HDMF was lowest at pH values between 4 and 5. Thus, to demonstrate the enzymatic formation of HDMF, incubation experiments with *Z. rouxii* and strawberry protein extract were carried out at pH 5. The formation of enantiomerically enriched HDMF was demonstrated in both experiments whereas racemic furanone was detected under neutral pH conditions [72].

### 5.2. Bacteria

HDMF was detected after 4 days of growth of *Pichia capsulata* on casein peptone culture medium containing L-rhamnose [75]. Stable isotope ratio mass spectrometry analysis confirmed L-rhamnose as carbon source for HDMF. Time course experiments led to the hypothesis that HDMF is formed by *P. capsulata* from an intermediate which was generated during thermal sterilization of the culture medium as was proposed for yeast [63,64]. Similarly, HDMF was detected in media prepared with heated sugar and amino acids as a result of the Maillard reaction [76]. However, enhanced level of HDMF was observed in the same media fermented by *Lactococcus lactis* subsp. cremoris.

## 6. Formation in Plants

The biosynthesis of HDMF has been intensively studied in strawberry fruit and although the complete biosynthetic route has not yet been fully discovered, substantial progress has been achieved. Radiotracer studies identified carbohydrates as the natural precursors of HDMF and its derivatives [77]. Incorporation rates of 1-^3^H-D-glucose, U-^14^C-D-glucose, U-^14^C-D-glucose-6-phosphate, U-^14^C-D-fructose, and U-^14^C-D-fructose-1,6-diphosphate into the total amount of furanones were 0.032, 0.035, 0.147, 0.202, and 0.289% of the radioactivity entering the fruit, respectively. Deoxysugars, which are excellent precursors for HDMF in the Maillard reaction, were not transformed into furanones *in planta*. Later, D-fructose and D-fructose-6-phosphate were confirmed as progenitor of HDMF by an independent experiment [78].

The incorporation of the radioactivity of S-methyl-^14^C-adenosyl-L-methionine (^14^C-SAM) and chemically synthesized 2-(or 5-)methyl-^14^C-HDMF [79] into the furanone structures supported the conclusion that ^14^C-SAM is the source of the methyl group in the 4-methoxy compound DMMF and that HDMF is the precursor of DMMF and HDMF glycosides [13]. This observation led the characterization of an enzymatic activity in strawberry protein extracts that transferred a methyl group from SAM to HDMF and sharply increased during ripening of strawberry fruits [80]. The partially purified enzyme had a native molecular mass of 80 kDa, with optimum activity at pH 8.5 and a high apparent K_m_ value of 5 mM for HDMF whereas 1,2-diphenolic such as caffeic acid and protocatechuic aldehyde were accepted with much higher affinities (K_m_ 130 and 20 µM, respectively). Cloning of the corresponding cDNA and characterization of the encoded protein provided the nucleotide sequence of *Fragaria x ananassa* *O*-methyltransferase (*FaOMT*) and confirmed the methylation of HDMF to DMMF by a SAM-dependent OMT [81]. Transcript analysis indicated the accumulation of *FaOMT* mRNA during ripening in strawberry fruit. Transcipts were absent in root, petiole, leaf and flower. A common feature of the accepted substrates was an o-diphenolic structure also present in HDMF in its dienolic tautomer. Due to its expression pattern it was supposed that FaOMT is involved in lignification in the expanding fruit and the biosynthesis of DMMF during strawberry fruit ripening. The dual function was further substantiated by transformation of strawberry with the *FaOMT* sequence in sense and antisense orientation, under the control of the cauliflower mosaic virus 35S promoter [82]. The repression of *FaOMT* resulted in a near total loss of DMMF, whereas the levels of the other volatiles remained unchanged. Furthermore, the ratio of feruloyl 1-*O*-β-D-glucose and caffeoyl 1-*O*-β-D-glucose was affected, indicating a dual function of the enzyme *in planta*. Only recently, while investigating the genetic factors controlling fruit flavor *FaOMT* was identified as the locus controlling natural variation in DMMF content [83]. Sequence analysis identified 30 base pairs in the promoter of an inactive *FaOMT* homoeolog containing putative binding sites for basic/helix-loop-helix, MYB, and BZIP transcription factors. This polymorphism co-segregated with both the presence of DMMF and the high expression of *FaOMT* during ripening.

**Scheme 2:**
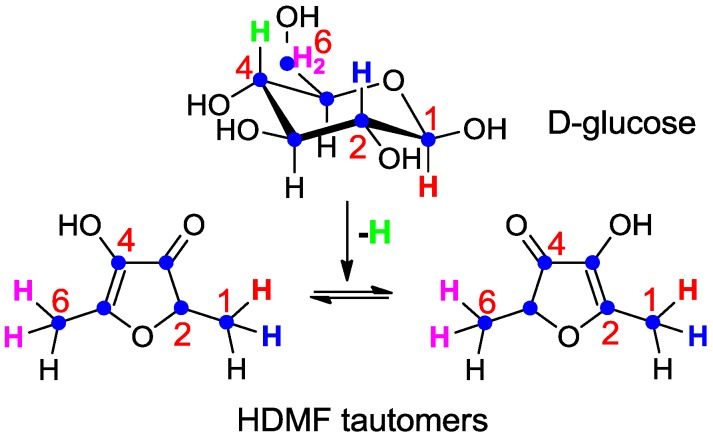
Metabolic fate of isotopes during the transformation of D-glucose to the HDMF tautomers (summarized from [85]). The complete carbon chain (blue dots) of D-glucose was recovered in HDMF. Hydrogen atom (green) located in position 4 of D-glucose was lost during the transformation whereas hydrogen atom (blue) at position 2 in D-glucose was shifted to carbon 1 in HDMF. Hydrogen atoms located at position 1 (red) and 6 (pink) in D-glucose remained attached to the same carbon in HDMF.

Although radiotracer experiments identified D-fructose-1,6-diphosphate as the most efficient precursor of HDMF in strawberry fruit [77] the localization of the label in HDMF could not be unambiguously determined due to the keto-enol tautomerism and the instability of HDMF. Thus, D-fructose labeled with stable isotopes (1-^13^C-D-fructose and U-^13^C_6_-D-fructose) was applied to detached ripening strawberry fruits, and the incorporation into the furanones determined [84]. The data proved the direct conversion of D-fructose to the furanones without cleavage of the carbohydrate skeleton prior to the formation of the furanones, as expected for a biological Maillard reaction. The label was primarily incorporated into the furanone moiety of the HDMF glycosides, indicating that D-fructose is a more efficient precursor of the furanone than D-glucose component of the HDMF glycosides. More elaborate experiments using different isotopically labeled carbohydrates clearly showed that 1- and 6-deoxy-D-glucose, (-D-fructose) are not natural precursors of the furanones [85] and thus confirmed the data obtained by radiotracer studies [77]. However, 1-^2^H-, 2-^2^H-, 6,6-^2^H_2_-D-glucose as well as U-^13^C_6_-, 1-^13^C-, 1-^2^H- and 6,6-^2^H_2_-D-fructose were transformed to the furanones (Scheme 2). The complete carbon chain of the carbohydrates was recovered in HDMF whereas the isotope label of 4-^2^H-D-glucose was not found in the furanones. The observed isotope shift from 2-^2^H-D-glucose to 1-^2^H-HDMF (Scheme 2) can be explained by the catalysis of phosphohexose isomerase in the course of the biogenesis of the furanones [85]. Thus, D-glucose is metabolized to D-fructose-6-phosphate prior to the transformation into HDMF.

**Scheme 3:**
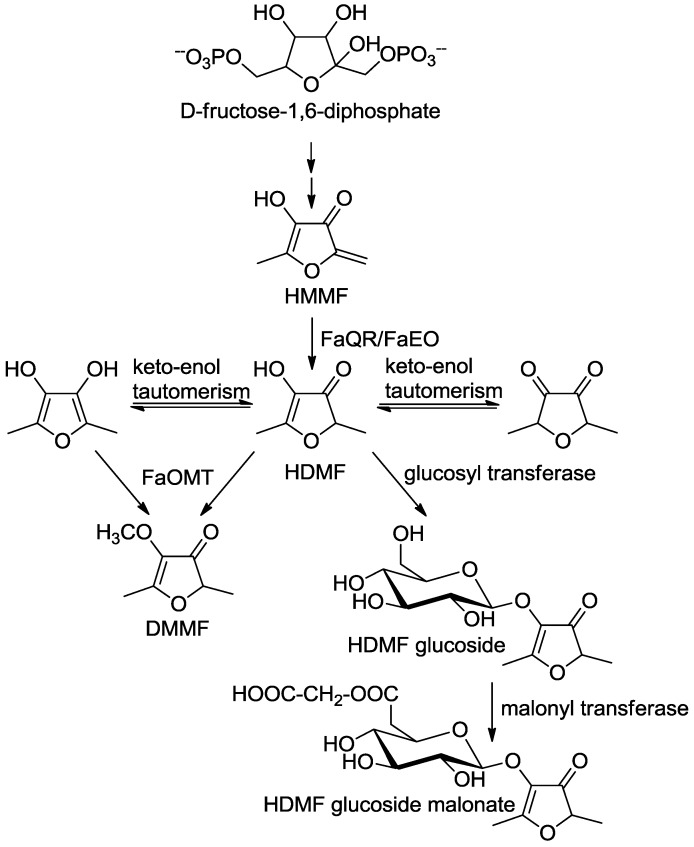
Postulated biosynthetic pathway of furanones in strawberry fruit (adapted from [86]). 4-Hydroxy-5-methyl-2-methylene-3(2*H*)-furanone, HMMF; 4-hydroxy-2,5-dimethyl-3(2*H*)-furanone, HDMF; *Fragaria x ananassa* quinone oxidoreductase, FaQR [86]; *F. x ananassa* enone oxidoreductase FaEO [87]; *F. x ananassa* *O*-methyltransferase FaOMT [80,81,82].

Next, an enzyme involved in the biogenesis of HDMF was partially purified. The observed distribution of enzymatic activity correlated with the presence of a single polypeptide [86]. Sequence analysis showed total identity with the protein sequence of a ripening-induced, auxin-dependent putative quinone oxidoreductase (FaQR). The FaQR protein was functionally expressed in *Escherichia coli* and catalyzed the formation of HDMF. 4-Hydroxy-5-methyl-2-methylene-3(2*H*)-furanone (HMMF) was identified as natural substrate of FaQR and precursor of HDMF (Scheme 3, [86]).

As FaQR catalyzes the reduction of the α,β-unsaturated bond of the highly reactive enone HMMF it was later renamed to *F. x ananassa* enone oxidoreductase (FaEO; [87]). FaEO does not reduce the double bond of straight-chain 2-alkenals and 2-alkenones but rather hydrogenates a number of HMMF derivatives substituted at the methylene functional group. HMMF was also detected in tomato and pineapple fruit which suggested that HDMF is synthesized in different fruits by a similar pathway [87]. *Solanum lycopersicon* EO (*SlEO*) was cloned from cDNA and the recombinant protein characterized. Biochemical studies confirmed the involvement of *SlEO* in the formation of HDMF in tomato fruit [87]. FaEO and SlEO exhibited a narrow substrate spectrum in comparison to two other NAD(P)H-dependent non-flavin ene reductases [88]. Only recently, to elucidate the molecular mechanism of the peculiar reaction catalyzed by FaEO, its crystal structure in altogether six different states or complexes, including those with HDMF as well as with three substrate analogs was determined [89]. The data revealed that the 4R-hydride of NAD(P)H is transferred to the unsaturated exocyclic C-6 carbon of HMMF, resulting in a cyclic optically inactive enolate intermediate that subsequently becomes protonated, eventually producing HDMF [89].

Notably some reports suggested that the production of furanones may not be a straightforward activity of a plant metabolite pathway alone, but a combined effort of the strawberry plant and an associated bacterium, *Methylobacterium extorquens* [4,90]. However, this proposed route is not convincing as contradictory reports were published about the final steps to HDMF and DMMF and tracer experiments do not support the transformation of the proposed intermediates lactaldehyde and 6-deoxy-D-fructose-1-phosphate to the furanones [77,85].

## 7. Conclusions

The 3(2*H*)-furanones are important aroma chemicals due to their low odor thresholds and attractive flavor properties. They are chemically formed from different carbohydrates during the Maillard reaction and thus occur in a number of processed foods where they contribute to the aroma. However, furanones are also produced by yeast, bacteria and plants and show different physiological functions which are probably due to their redox activity. Although deoxysugars such as L-rhamnose are efficient precursors of HDMF in the Maillard reaction, D-fructose-1,6-diphosphate was identified as the natural progenitor in fruit. In strawberry fruit, the phosphorylated carbohydrate is converted by phosphate and water elimination to HMMF which is eventually reduced by FaEO (FaQR) to HDMF. Methylation of HDMF leads to the accumulation of DMMF and is catalyzed by FaOMT. Overall, substantial progress has been made towards the elucidation of the biosynthetic pathway of natural furanones in micro-organisms and plants which was made possible by the application of isotopically labeled precursors. In the near future, the knowledge of the genome sequence of the woodland strawberry [91] will enable the detection of the missing genes of the HDMF pathway and improved imaging systems [92] can help to locate the furanones intracellular. Knowledge of the complete set of involved genes and enzymes would provide the foundation for the biotechnological production of natural furanones.
